# Progression of electrophysiological impairments in diabetic cardiomyopathy and intervention using *Enicostemma axillare*

**DOI:** 10.55730/1300-0152.2733

**Published:** 2024-11-04

**Authors:** Victor Arokia DOSS, Dharaniyambigai KUBERAPANDIAN

**Affiliations:** Department of Biochemistry, PSG College of Arts & Science, Tamil Nadu, India

**Keywords:** Blood glucose, diabetes, cardiac hypertrophy, diabetic cardiomyopathy, electrocardiography

## Abstract

Earlier studies widely reported advanced ventricular impairments only at the later stages of diabetic cardiomyopathy (DCM). The present study aimed to understand the duration-based early to late stages of cardiac electrophysiological impairments using electrocardiography (ECG) and intervention using *Enicostemma axillare* subsp. *littorale*. The experimental rats were streptozotocin (SZ)-induced diabetic rats that were administered *E. axillare* formulations as follows: (1) normal; (2) SZ - 40 mg/kg, single, i.p.); (3) SZ + insulin (2 U/day) + losartan (10 mg/kg); (4) SZ + insulin + losartan + *E. axillare* decoction (2 mL/day); (5) SZ + *E. axillare* decoction; (6) SZ + *E. axillare* (low dose-500 mg/kg), and (7) SZ + *E. axillare* (high dose-2 g/kg). Steady hyperglycemia was witnessed until day 5 followed by various elevated patterns until day 60 in the SZ group that was potentially treated with *E. axillare* formulations from day 20. ECG (lead II) revealed early significantly impaired ventricular events, namely widened QRS complex, elevated R-amplitude, and prolonged R–R interval from day 10 that were regulated using *E. axillare* decoction. Pearson’s correlation analysis revealed a strong relationship between basal blood glucose (day 0) and impaired ECG parameters. This duration-based study therefore illustrated the progression of glycemic shifts, ventricular impairments, and their correlation besides establishing the interventional potential of *E. axillare* subsp. *littorale* towards these impairments associated with DCM. These glycemic-ECG impairments were substantiated by the increased blood HbA1c, serum NT-pro BNP, and LDH levels that were ameliorated by *E. axillare* subsp. *littorale* decoction. It was concluded that *E. axillare* subsp. *littorale* can restore early ventricular depolarization impairments, marking it as the reversible hypertrophic cardiomyopathic stage towards intervention in DCM.

## 1. Introduction

Diabetes is expected to affect 640 to 700 million people by 2045 and is associated with 30% and 8% cardiac risks in type 1 diabetes mellitus (T1DM) and type 2 diabetes (T2DM), respectively. Diabetic cardiomyopathy (DCM) is defined as structural and functional impairments of the heart, preferably left ventricular enlargement (cardiac hypertrophy (CH)) in its initial stages that advances into dilated ventricles (dilated cardiomyopathy) leading to heart failure. It occurs due to several cross-linked metabolic shifts and inflexibility driven exclusively by high blood glucose levels even in the absence of other comorbidities like hypertension or other coronary diseases. DCM develops as a consequence of chronically sustained metabolic adaptation for cellular stress, thereby causing impairments in the electrophysiological events in cardiac muscles. This is followed by the structural accumulation of extracellular matrix (ECM) proteins like collagen, termed cardiac fibrosis (CF). These lead to ventricular wall stiffening and diastolic dysfunction, ultimately causing heart failure, asymptomatically (Jia et al., 2018; Filardi et al., 2019; De Blasio et al., 2020; Kuberapandian and Doss, 2021; Heather et al., 2022; Huo et al., 2023).

Electrocardiography (ECG) depicts primary cardiac changes (Stern and Sclarowsky, 2009; Kuberapandian and Doss, 2023) but mostly cardiac dysfunction is clinically diagnosed mostly only at later stages using echocardiography or other imaging techniques (Miki et al., 2013). Previous studies widely reported treatment of glucose-cardiac impairments by various therapeutic compounds and herbs only at the advanced stages with less focus on the early events underlying DCM progression (Tian et al., 2017).

*Enicostemma axillare* subsp. *littorale*, commonly known as Indian Gentian (Vellarugu in Tamil and Mamejava in Ayurveda; Family - Gentianaceae), is a potent antidiabetic and antihypertrophic medicinal herb with enormous pharmacological properties due to its unique phytocompound, swertiamarin (Saranya et al., 2013; Li et al., 2016; Doss and Kuberapandian, 2021). In the present study it was hypothesized that screening of cardiac-ECG parametric changes from the initial hyperglycemic stage in diabetes through duration approaches is essential for a better understanding of DCM pathophysiology and its intervention at the primary stage (Nielson and Lange, 2005; De Blasio et al., 2020; Marchini et al., 2020). Hence, the aim was to screen the sequential changes in the blood glucose levels and the ventricular-ECG parameters at different durations of diabetes to DCM stages besides evaluating the interventional potential of *E. axillare* towards the same.

## 2. Materials and methods

### 2.1. Chemicals and kits

All chemicals and reagents used were analytical grade from Hi Media Pvt Ltd., India. Streptozotocin was purchased from SRL Pvt. Ltd., India. Insulin (human insulin) was purchased as NovoPen 4 (Novo Nordisk, Catalog No: 8-4253-00-013-1), and losartan (Losar 25) and an Accu-Check Instant S blood glucose meter with strips (Roche) were purchased from a local pharmacy. A whole blood HbA1c determination kit was purchased from Agappe Diagnostics (Catalog No: 12011042). Serum N-terminal pro-brain natriuretic peptide (NT-pro-BNP) was estimated using a rat NT-proBNP chemiluminescence immunoassay (CLIA) from AssayGenie Technologies (Catalog No: RTES00412). Serum lactate dehydrogenase (LDH) was assayed using an Autospan Liquid Gold LDH kit (Catalog No: 74LS100-25). *Enicostemma axillare* subsp. *littorale* decoction and plant powder extracts were prepared as shown below.

### 2.2. Preparation of *E. axillare* subsp. *littorale* decoction and powder extracts

*Enicostemma axillare* subsp. *littorale* (Blume) A.Raynal was authenticated by the Botanical Survey of India, Southern Regional Centre, Coimbatore (BSI/SRC/5/23/2010-11/Tech-2051 and BSI/SRC/5/23/2023-24/Tech-517). The decoction (kwatha formulation) was prepared as per Ayurvedic formulations (Raj et al., 2018). Initially 300 g of clean shade-dried coarse plant powder (whole plant containing vegetative and reproductive parts) was soaked in 5 L of distilled water for 15 h with maceration. Next it was heated at 80–90 °C and the volume was reduced to 300 mL (1/16 volume), which was used for oral administration in rats. Concomitantly, the powder forms were prepared by macerating the whole plant powder in distilled water (1:10) for 72 h with intermittent shaking and filtering, and the concentrate was dried under reduced pressure in a temperature controlled (80–90 °C) water bath to yield brown powder extracts (Raj et al., 2018; Doss and Kuberapandian, 2021), which were administered to rats in low and high doses.

### 2.3. Experimental rats

Male Sprague Dawley rats aged 60 to 70 days postnatal (young adult stage) weighing between 140 and 200 g (n = 4 per group) (Al-Fahham, 2018) were procured after ethical clearance (CPCSEA/No:399/2018/IAEC) (n = 32). The sample size (n) per group was determined through the resource equation method and the sample size for repeated measures ANOVA (Zhong, 2009; Charan and Kantharia, 2013). The rats were acclimatized for 3 days under controlled temperature of 29 ± 5 °C, humidity at 55% ± 5, and 12 h light/dark cycles. The following experimental conditions were used:

**Group I –** normal (control - NOR) received 0.1 M citrate buffer (pH 4.5)
**Group II –** streptozotocin (SZ-40 mg/kg, i.p., single dose) dissolved in 0.1 M citrate buffer (pH 4.5) (Mostafavinia et al., 2016)
**Group III –** SZ + insulin (2 U/day, s.c.) (Badole et al., 2015) + losartan (10 mg/kg, oral) (Doss and Kuberapandian, 2021)
**Group IV –** SZ + insulin (2 U/day, s.c.) + losartan (10 mg/kg, oral) + *E. axillare* subsp. *littorale* decoction (2 mL/day, oral) (Raj et al., 2018)
**Group V –** SZ + *E. axillare* subsp. *littorale* decoction (2 mL/day)
**Group VI –** SZ + low dose *E. axillare* subsp. *littorale* (500 mg/kg, oral) (Doss and Kuberapandian, 2021)
**Group VII –** SZ + high dose *E. axillare* subsp. *littorale* (2 g/kg, oral) (Saranya et al., 2013).

### 2.4. Glycemic index in experimental rats

The experimental rats were screened for fasting blood glucose measurements by Accu-Check Instant S glucometer at regular intervals for 60 days (Saad et al., 2015).

### 2.5. Electrocardiography (ECG) analysis of CH underlying DC

To assess the impact of impaired blood glucose levels on cardiac electrophysiological events in vivo, the conventional bipolar limb lead II, which is the fundamental ECG analysis for ventricular hypertrophy, was screened using BITalino ECG Sensor - OpenSignals [r]evolution software for 6 min in unanesthetized rats (Doss and Kuberapandian, 2021). The changes in nine ECG parameters, namely P-wave (ms and mV), P–R interval (ms), QRS complex (ms), R amplitude (mV), ST segment (ms and mV), R–R interval (ms) and pulse/heart beat rate (HR in bpm), were assessed before (day 0) and in due course for 60 days.

### 2.6. Diabetic and cardiac markers

After ECG recordings at the end of 60 days, the experimental rats were subjected to cardiac puncture to collect blood for serum preparation and blood was also placed in an anticoagulant-EDTA coated tube for estimating HbA1c. Next the rats were sacrificed under anesthesia using mild chloroform (Doss and Kuberapandian, 2016; Doss and Kuberapandian, 2021) for further investigations. The whole blood HbA1C was estimated using a Vector Biotec Biochemistry analyzer (Engbaek et al., 1989). Determination of NT-proBNP in the serum of the experimental rats was performed using a Tosoh Chemiluminescence immunoassay (CLIA) plate analyzer (Pfister et al., 2002). Determination of serum lactate dehydrogenase (LDH) was performed spectrophotometrically (Buhl and Jackson, 1978).

### 2.7. Data analysis

All the data obtained were expressed as mean ± standard deviation (SD) (n = 4 per group) from two replicates (duplicates) per sample. The data were verified statistically using two-way ANOVA in IBM SPSS 26 with a significance level of p < 0.05. Pearson’s correlation was performed in MetaboAnalyst 5.0 for the different durations of blood glucose and four ventricular hypertrophic ECG parameters (QRS complex, R-amplitude, ST segment amplitude, and R–R interval) to understand the relationship between them (Kuberapandian and Doss, 2021). Two-way ANOVA was performed for the seven experimental groups and durations as independent variables with blood glucose, body weight, and the nine ECG parameters as dependent variables. Multiple comparisons were performed using Scheffe’s post-hoc analysis to screen significant differences between and within experimental groups and durations whose significance were indicated by an asterisk respectively (Ananthakrishnan and Doss, 2013; Dhakal, 2019) as shown in Tables 1 and 2.

## 3. Results

### 3.1. Sequential glycemic shift among the experimental groups

**Figure 1:**
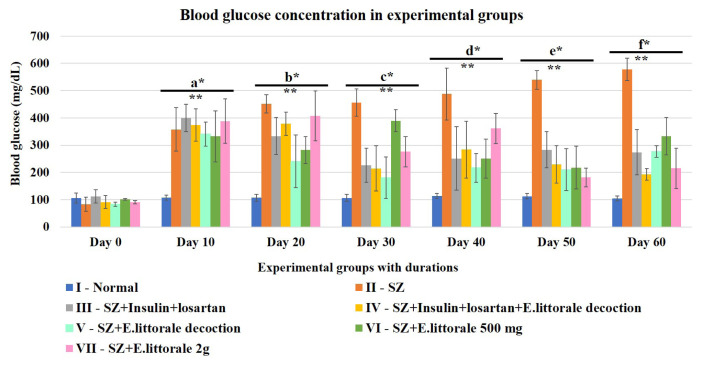
Chronic hyperglycemia at various durations. SZ rats (group II) exhibited more significant hyperglycemia than normal rats (group I). All the treated groups (III to VII) especially with *E. axillare* subsp. *littorale* decoction significantly reduced this elevation preferably from day 20 onwards in group VI. Two-way ANOVA for early durations of blood glucose showed significant relationship between experimental groups and the durations [F (78,294) = 9.805, p = 3.447E-48] at every 10 day-interval [F (36,147) = 6.514, p = 1.1153E-16]. This indicated that the efficacy of the experimental conditions varies in different durations based upon the respective potentials towards induction or treatment of hyperglycemia.

The blood glucose levels significantly rose steadily within 2 days of SZ administration in all the experimental groups except for the *E. axillare* subsp. *littorale* decoction (group V) administered rats. Five different glycemic shift patterns were observed in the diseased (SZ-group II) rats (Supplementary Figure 1; Supplementary Table 1). Though the glucose levels seem mildly decreased from day 3, the SZ rats (group II) continued to show hyperglycemia until day 60 (Figure 1 and Supplementary Figure 1; Supplementary Table 1). After day 10, the groups administered *E. axillare* decoction (groups IV and V) and the groups administered the low and high doses of extract powder (groups VI and VII) exhibited reduced blood glucose levels similar to the reference drug (group III).

### 3.2. Body weight among the experimental groups

The SZ (group II) rats showed increased body weight (BW) initially up to day 30 and a steady decrease was observed from day 50 (Supplementary Figure 2). Though the changes in BW were not highly significant among the experimental groups, over the duration it was seen that the *E. axillare* subsp. *littorale* decoction (group V) administered rats displayed significant increases in BW (Supplementary Figure 2).

### 3.3. Electrocardiographic (ECG) analysis of cardiac events

**Figure 2:**
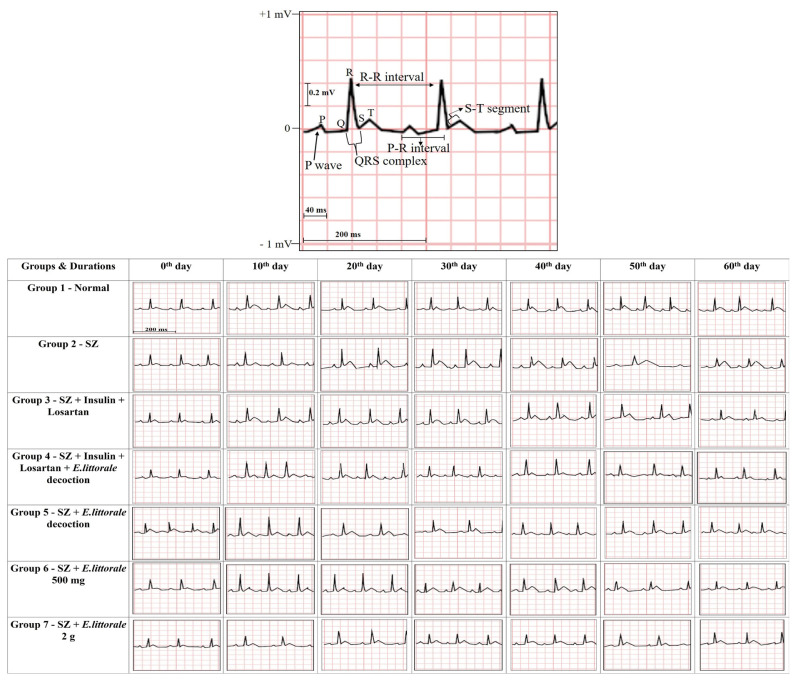
Graphical representation of ECG recordings at various durations. P wave: atrial depolarization; P–R interval: atrioventricular propagation of impulse; QRS complex and R-amplitude: ventricular depolarization; ST segment: ventricular repolarization; R–R interval: duration between two heart beats/heart rate (HR).

**Figure 3:**
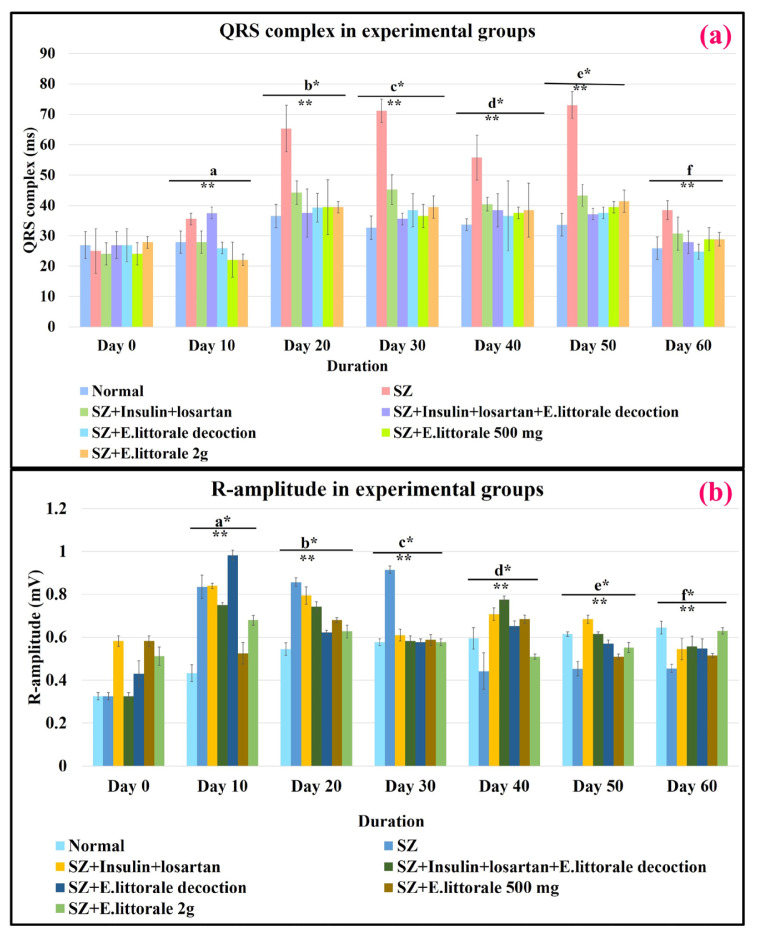
ECG parameters of ventricular depolarization. Delayed communication between ventricular cardiomyocytes was significantly observed by widened QRS and elevated R-amplitude from day 20 in diseased (SZ-group II) rats that were regulated by *E. axillare* subsp. *littorale* formulations. Two-way ANOVA for the QRS complex [F (36,147) = 6.092, p = 1.43E-15] and R-amplitude [F (36,147) = 61.581, p = 7.6532E-72] showed significant interactions between experimental conditions (groups) and durations that can influence the ventricular events (QRS complex and R-amplitude) together. This indicated that the experimental conditions and different durations are associated with changes in QRS complex and R-amplitude.

**Figure 4:**
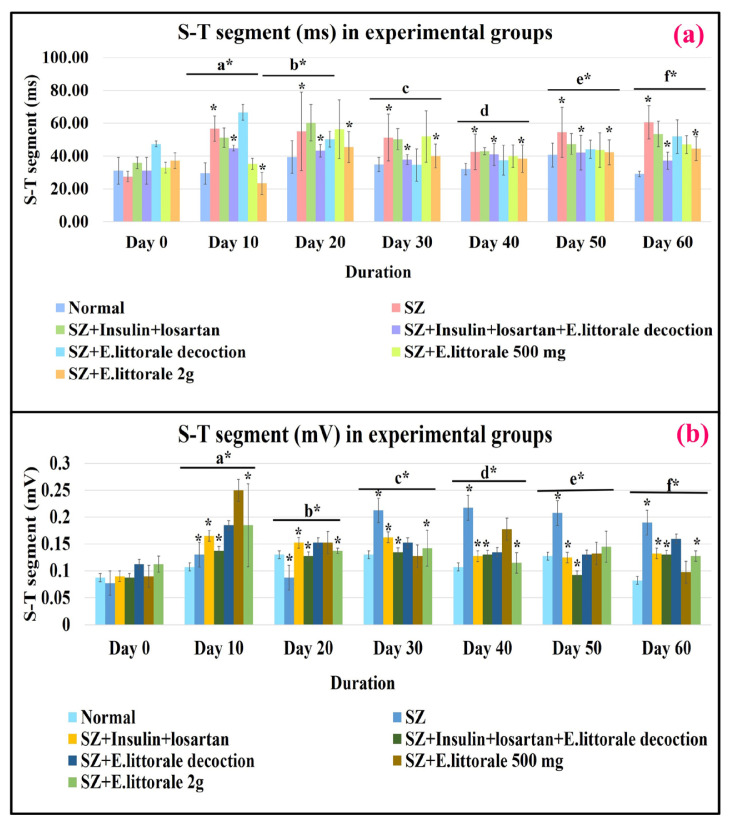
ECG parameters for ischemic conditions. The disease group (SZ-group II) exhibited a significantly elevated ST segment (ms and mV) indicating the presence of ischemia and impaired ventricular events that were significantly reduced by coadministration of *E. axillare* subsp. *littorale* decoction with insulin and losartan (group IV). Relationships between experimental groups and durations were significant for ST segment (ms) [F (36,147) = 2.638, p = 0.000024] and ST segment (mV) [F (36,147) = 8.436, p = 2.4215E-21]. This indicated that the experimental conditions and durations can influence the ST segment.

**Figure 5:**
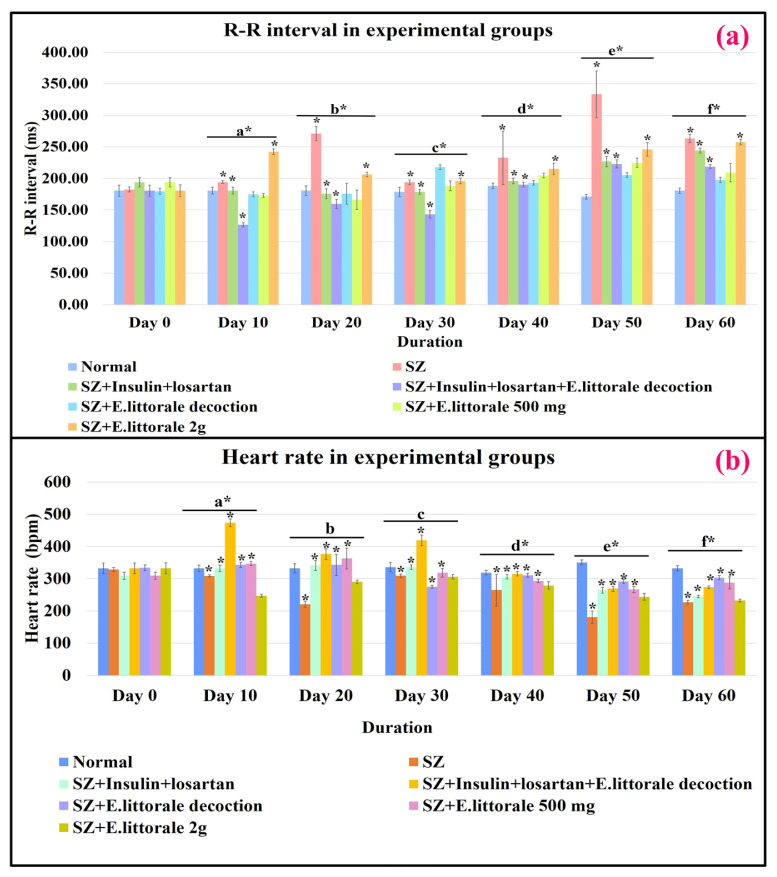
ECG parameters of heart rhythm. The disease group (SZ-group II) was observed with prolonged R–R interval embarking reduced HR (bradycardia) that were extended until day 60 and were under control in the treatment groups. Statistically significant interactions between experimental groups and durations were seen in R–R interval (ms) [F (36,147) = 8.438, p = 2.4215E-21] and heart rate [F (36,147) = 25.565, p = 4.5217E-47]. This shows that both experimental groups and durations have significant effects upon the changes in R–R interval and HR.

The respective ventricular electrophysiological events (lead II) and the shifts in respective ECG parameters of the experimental groups are represented in Figures 2 to 5 and Supplementary Figures 3 and 4. Overall, (i) significant shifts in the atrial events such as P-wave (ms and mV) were observed only in the reference drug group (group III) from days 10 to 60. (ii) Similarly, significant differences in atrial/ventricular communication (P–R interval) were observed only on day 10. (iii) Impaired ventricular electrophysiological events were observed from day 10 marked by the initiation of elevated R-amplitude (mV), widened QRS complex, and mildly impaired ST segment (ms and mV), thereby impacting R–R interval until day 30 in the SZ administered rats (group II) when compared to the normal rats.

#### 3.3.1. Ventricular depolarization

From day 40 the height of R-amplitude (mV) decreased accompanied by significant widening of the QRS complex (ms) and extremely prolonged R–R interval (ms), which on day 50 was followed by an elevated ST segment (mV). Significant restoration of QRS complex and R-amplitude to near normal were observed from day 20 in groups treated with *E. axillare* subsp. *littorale* formulations (groups IV to VII) when compared to the reference drug (group III) (Figures 2, 3a, and 3b; Supplementary Tables 2 and 3).

#### 3.3.2. Ventricular repolarization

An elevated ST segment (ms and mV) was observed from day 10 to 60 in the SZ administered groups with a sudden fall but positively deflected ST segment (mV) on day 20 that were regulated by *E. axillare* subsp. *littorale* formulations (Figures 2, 4a, and 4b; Supplementary Table 4).

#### 3.3.3. Cardiac rhythm

Significantly mild to drastic impairments in R–R interval and HR were observed across all durations (days 10 to 60), especially during the early period (before day 30). These were potentially restored in groups coadministered *E. axillare* subsp. *littorale* decoction and reference drugs (group IV) besides the extract powder high dosage (group VII). Beyond day 40, a steady drastic dip was witnessed in the HR of disease control rats (SZ-group II) that were in groups treated with *E. axillare* subsp. *littorale* formulations. This overall displayed a profound effect in maintaining the HR to near normal level (Figures 2, 5a, and 5b; Supplementary Table 5).

Therefore, administration of *E. axillare* subsp. *littorale* decoction (group V) was an enormous intervention against widening of the QRS complex (ms) and impairment of R-amplitude (mV). Coadministration of *E. axillare* subsp. *littorale* decoction along with insulin and losartan (group IV) exhibited better restoration of cardiac events than in the group that was treated only with reference drugs (group III).

### 3.4. Biochemical markers of diabetes and cardiac hypertrophy

**Figure 6:**
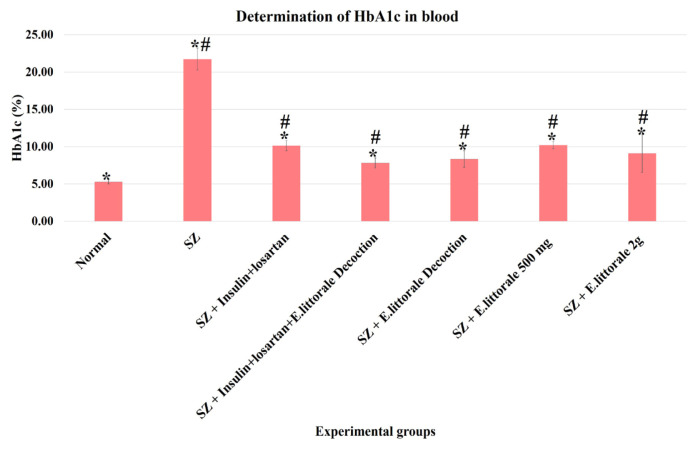
Determination of blood HbA1c in the experimental groups. A significantly increased HbA1c level was observed in the diseased group (SZ-group II) when compared to the normal group (group I). With comparison to the reference drugs (group III) the group coadministered *E. axillare* decoction and reference drugs (group IV) was highly efficient with reduced HbA1c levels. This was followed by the groups administered *E. axillare* decoction (group V) and low dosage extract powder (group VI).

**Figure 7:**
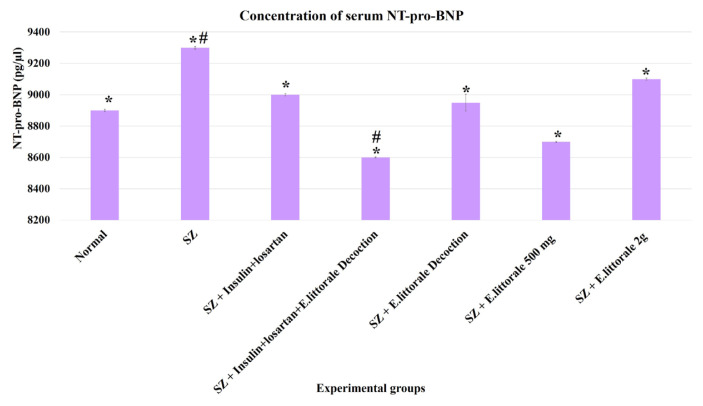
Determination of serum NT-pro BNP in the experimental groups. Significantly increased serum NT-pro BNP level was observed in the diseased group (SZ-group II) when compared to the normal group (group I). With comparison to the reference drugs (group III) the efficacy of the plant formulations in reducing NT-pro BNP levels to near normal levels was observed to be high in the group administered *E. axillare* decoction (group V).

**Figure 8:**
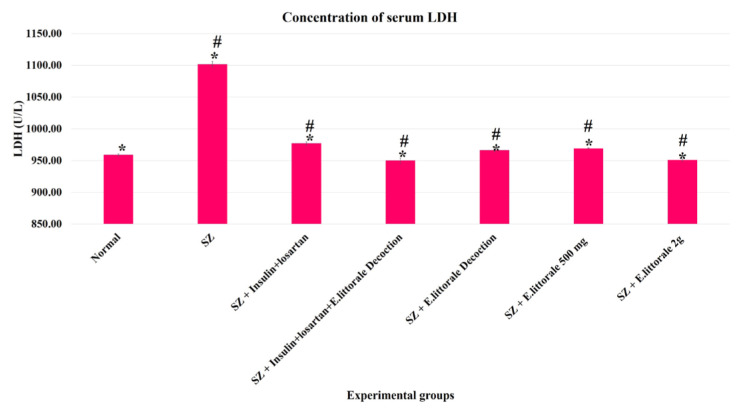
Determination of serum LDH in the experimental groups. Significantly increased serum LDH level was observed in the diseased group (SZ-group II) when compared to the normal group (group I). With comparison to the reference drugs (group III) the efficacy of the plant formulations in regulating LDH levels to near normal levels was observed to be highly potent in the groups treated with *E. axillare* decoction (groups IV and V) followed by the high dosage extract powder (group VII).

In the present study, the blood HbA1c levels were >20% in the SZ group (group II), whereas the groups administered *E. axillare* were observed to show an alteration in this shift (Figure 6). Similarly, elevated serum NT-pro BNP and LDH were observed in the SZ group (group II) but were absent in the groups administered *E. axillare* decoction (Figures 7 and 8). Coadministration of *E. axillare* decoction and reference drugs (group IV) displayed better efficacy than the reference drug (group III).

### 3.5. Shifts in blood glucose is not a direct inducer of ventricular impairments

Pearson’s correlation herein suggested that the strong positive relationship of basal blood glucose (day 0) indicates that the changes in glycemic levels (days 10 to 60) were not directly linked to ventricular impairments. In other words, it is the association of blood glucose with several multiple crucial factors and pathways (Miki et al., 2013; Jia et al., 2018; Filardi et al., 2019; De Blasio et al., 2020; Kuberapandian and Doss, 2021) that triggers the DCM progression and not blood glucose itself directly (Supplementary Figure 4).

### 3.6. Order of ventricular impairments in DCM and its stages of severity

**Figure 9:**
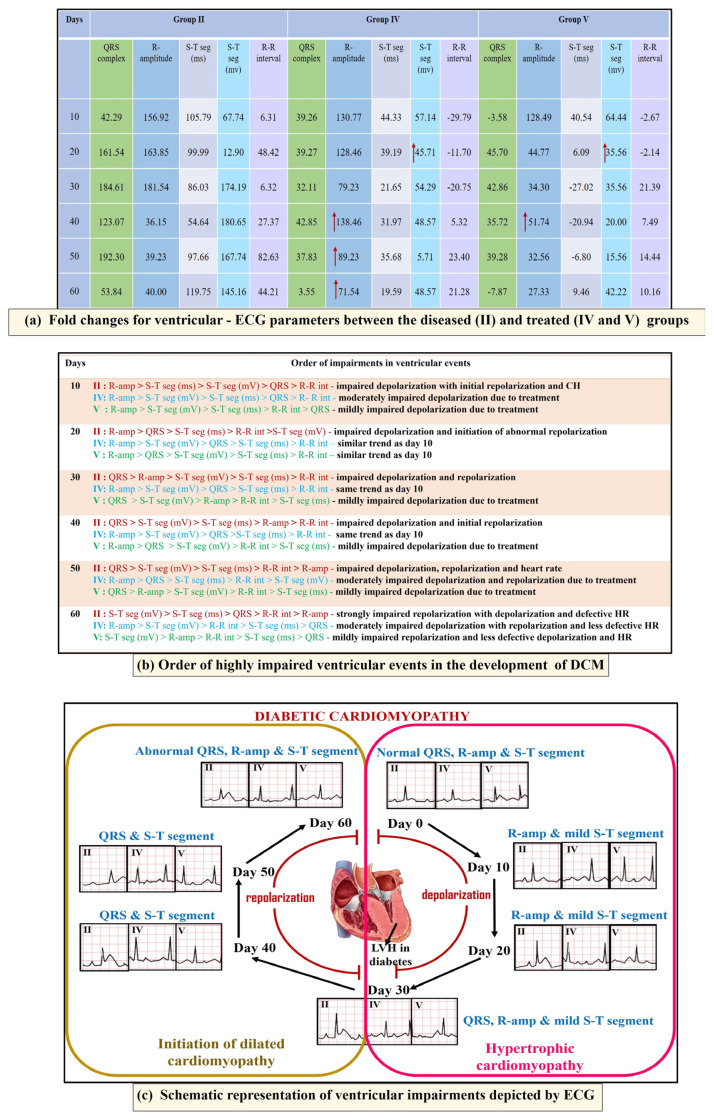
Overview of ECG parameters in DCM and the effect of *E. axillare* subsp. *littorale*. In the diseased group (SZ-group II) impaired ventricular depolarization (R-amplitude) was witnessed during the early days until day 30 embarking on hypertrophic state. Next, it was accompanied with widened QRS complex and elevated ST segment towards day 60, indicating dilated ventricles. Direct and coadministration of *E. axillare* decoction (groups IV and V) displayed a profound interventional effect upon these early ventricular hypertrophic impairments, thus preventing the progression of DCM.

Two groups, namely those coadministered the reference drug with *E. littorale* decoction (group IV) and *E. littorale* decoction (group V), exhibited potent interventions regarding the impairments in cardiac events when compared to the SZ disease control (group II) (Figure 9). The fold changes for each ECG parameter between groups (Figure 9a) were applied to find the ventricular status (Figure 9b). Ventricular impairments mildly began on day 10, progressed between days 20 and 30, representing hypertrophic cardiomyopathy, and further defective beyond day 40 (Figure 9c), indicative of the initiation of dilated cardiomyopathy.

## 4. Discussion

Many previous investigations reported the pathophysiology of DCM only after 8 to 12 weeks post-SZ with less focus on the early stages of DM to DCM transformation. Attempting to understand those sequential changes in blood glucose and the cardiac electrophysiology can aid in early and precise treatments and diagnosis of DCM (Gupta et al., 2018; Hegab, 2018; Jia et al., 2018; De Blasio et al., 2020; Marchini et al., 2020; Lezoulac’h et al., 2023).

In the present study, *E. axillare* subsp. *littorale* decoction and its coadministration with insulin and losartan exhibited an enhanced therapeutic effect, hereby recommending the use of this plant for a synergistic effect of marketed drugs. Flavonoids, phenols, terpenes, carotenoids, lycopenes, isoflavones, polyphenols, iridoid glycoside, and aliphatic alcohols were previously reported in *E. axillare* subsp. *littorale* (Lipinski, 2011; Ramesh and Dharaniyambigai, 2012; Saranya et al., 2013). These phytochemicals besides preventing body weight loss (VanHoose et al., 2010; Eleazu et al., 2013) could have also attributed towards regulating the SZ induced blood glucose levels (>200 mg/dL) (Figure 1). Therefore, the herb altered SZ mimicked type 1 diabetes associated impaired hypertrophic cardiac electrical impulse conduction in humans (Saad et al., 2015; Hegab, 2018; Jia et al., 2018; Marchini et al., 2020; Doss and Kuberapandian, 2021; Lezoulac’h et al., 2023). Most cardiac investigations in rodents reported limb lead II as a fundamental ECG parameter for screening CH (Sysa-Shah et al., 2015; Konopelski and Ufnal, 2016; Doss and Kuberapandian, 2021) as followed in the present study (Figure 2).

### 4.1. Atrial events

Impaired P-wave (ms and mV) due to SZ were consequences of abnormal myocyte length associated with parasympathetic nervous function, mitral valve closure, diastolic mitral regurgitation, and atrial fibrillation (VanHoose et al., 2010; Konopelski and Ufnal, 2016; Gupta et al., 2018; Marchini et al., 2020). The present study is the first to report restoration of atrial events by *E. axillare* indicating improved left atrial-ventricular (LV) conduction after day 20.

### 4.2. Ventricular depolarization

Unlike the previous post-8 week research (Jia et al., 2018; De Blasio et al., 2020; Marchini et al., 2020) the present study identified significant impairments since day 20. This was prior to fibrosis that initiates after 1 to 2 weeks of SZ administration (Figures 2, 3a, and 3b; Supplementary Tables 2 and 3). *E. axillare* subsp. *littorale*, containing swertiamarin, is known to affect liver fibrosis via transforming-growth factor (TGF-β), rejuvenate pancreatic islets, regulate insulin signaling, β-adrenergic (β-AR) system, alleviation of oxidative stress, expression of peroxisome proliferator-activated receptors (PPARs) towards lipid oxidation, restoration of sodium/potassium channels (Na^+^/K^+^ ATPase), prevent apoptosis of cardiomyocytes, and ECM homeostasis. These could be the underlying mechanistic routes of *E. axillare* in regulating the potassium (K^+^) channel (Kv4.3) and its associated gene expression systems; fibroblasts triggered hyperpolarization activated cyclic nucleotide gated channels (HCN also called pacemaker channels), cellular gap junction system of connexin proteins (Cx43 and Cx45), and elongated cardiomyocytes from day 10 in the disease (SZ) group (Wang et al., 2020; Gallego et al., 2021; Hadova et al., 2021; Zayas-Arrabal et al., 2021). These eventually affected the defective ventricular depolarization, which otherwise leads to lethal fibrillations and arrhythmias (Dadheech et al., 2013; Konopelski and Ufnal, 2016; Srivastava et al., 2016; Doss and Kuberapandian, 2021; Gallego et al., 2021). Moreover, the shortened R-amplitude characteristic of ventricular dilation and loss of cardiomyocytes witnessed after day 30, indicating end-stage cardiac dysfunction (Finocchiaro et al., 2020), were also affected by *E. axillare*.

### 4.3. Ventricular repolarization

Regulation of an abnormal ST segment (ms and mV) (Figures 4a and 4b; Supplementary Table 4) is indicative of restored cell membrane function, ion channels (as in Brugada syndrome), intraventricular conduction, water–electrolyte balance, and hyperstimulation of β-AR and potassium levels (Konopelski and Ufnal, 2016; Gupta et al., 2018; Doss and Kuberapandian, 2021) by *E. axillare* subsp. *littorale* (Doss and Kuberapandian, 2021).

### 4.4. Cardiac rhythm

*E. axillare* restored R–R interval and HR, thereby affecting diabetic tachycardia and bradycardia (Figures 5a and 5b; Supplementary Table 5) (VanHoose et al., 2010; Gupta et al., 2018; Marchini et al., 2020) due to regulation of impaired glucose, insulin, and potassium levels, besides regulation of fasted versus fed states and activation of the autonomous nervous system (Gallego et al., 2012; Dadheech et al., 2013; Srivastava et al., 2016).

### 4.5. Diabetic and cardiac biomarkers

To substantiate the existence and outcomes of hyperglycemia and CH in experimental rats, the levels of blood HbA1c (Figure 6), serum NT-pro BNP (Figure 7), and LDH (Figure 8) were also monitored in the present study, all of which are associated with defective mitochondrial metabolism (Lichscheidt et al., 2022). The elevated HbA1c (Figure 6) in the SZ group indicated poor glycemic control (as witnessed above Figure 1). *E. axillare* subsp. *littorale* administration was found to significantly alter the HbA1c rise at the end of 60 days when compared to the diseased (SZ) group, hereby reestablishing its glycemic regulatory potential (Saranya et al., 2013; Sherwani et al., 2016; Srivastava et al., 2016).

Previous studies reported cardiac volume or pressure overload released NT-pro BNP levels (>300 pg/mL) to promote vasodilation, which is a primary, inexpensive clinical hypertrophic and myocardial inflammatory biomarker (Singla and Bhargava, 2016), wherein levels >1000 pg/mL were associated with a high risk of death. NT-pro BNP is associated with human diabetic heart triggered LVH, systolic dysfunction (LVSD), atrial fibrillation, and diastolic dysfunction (Fringu et al., 2020; Malachias et al., 2020; Malachias et al., 2022) characterized by P–R interval, QRS complex, R-amplitude, and ST segment as observed above in the present study. Previous STZ and DCM models have witnessed elevated NT-pro BNP at the end of 4 weeks (Hu et al., 2019; Zheng et al., 2019; Lin et al., 2020). The similar observation in the present study (60 days) hereby substantiates CH presence and this is the first report on the effect of *E. axillare* upon NT-pro BNP (Figure 7), which could be attributed to the aforementioned regulated mechanistic events that underlie ventricular depolarization warranting further investigations.

Similar to the elevated LDH levels in humans that initiate within 6 to 12 h peaking between 1 to 3 days of myocardial injury (Aydin et al., 2019), the STZ cardiomyopathic rat models displayed elevated serum LDH levels at the end of 6 weeks (Komolafe et al., 2014; Abukhalil et al., 2021). In the present study (Figure 8) at the end of 60 days, elevated LDH indicative of myocardial ischemia was substantiated by the elevated ST segment and widened QRS complex with elevated R-amplitude in ECG as a consequence of distorted myocardial architecture as discussed above. Although our previous study (Kuberapandian and Doss, 2021) and other studies (Bhatt et al., 2012; Selvam et al., 2018) reported various shifts in LDH levels, the precise mechanism beyond oxidative stress was not reported. In accordance with our previous study (Kuberapandian and Doss, 2021), the present study hereby postulates the interplay of metabolic remodeling by AMP-activated protein kinase (AMPK) and reactive oxygen species (ROS – oxidative stress) could underlie the impaired LDH levels during CH and DCM.

Therefore, the present study illustrated the sequential glycemic-electrophysiological shifts in diabetic rats starting at the end of day 60 (8 weeks) as the transition between preadvanced to advanced stages of DCM. This duration-based study in SZ rat models reflected chronic diabetic sugar levels of humans that can trigger cardiac impairments, which was fundamentally explored herein. As illustrated in Figure 9, our study highlights the restoration possibilities of cardiac events prior to advanced DCM stage if targeted in early stages that were screened through ECG during administration of *E. axillare* decoction and its coadministration with insulin and losartan. This is the first study to show the effect of *E. axillare* upon the hypertrophic marker NT-pro BNP. A limitation of the study, which can be taken into account in future research, is the extension of this investigation towards evaluating human glycemic-ECG changes of large samples and populations. Such clinical data can be further employed in several machine-learning-based artificial intelligence-dependent automation tools for early prediction and prognosis of DCM.

## Supplementary Data

**Supplementary Table 1:** Effect of experimental conditions on the levels of hyperglycemia during various durations.

Days	Hyperglycemic order	Blood glucose during diseased (mg/dL)	Blood glucose upon treatment (mg/dL)
**Day 10**	Reference drugs > EL2g > SZ > DD > ELD > EL500	>300	350–450
**Day 20**	SZ > EL2G > DD > reference drugs > EL500 > ELD	>400	250–300
**Day 30**	SZ > EL500 > EL2G > reference drug > DD > ELD	>400	150–350
**Day 40**	SZ > EL2G > DD > reference drug > EL500 > ELD	>450	250–450
**Day 50**	SZ > reference drug > DD > EL500 > ELD > EL2g	>500	150–280
**Day 60**	SZ > EL500 > reference drug > ELD > EL2g > DD	>550	190–310

Supplementary Table 1 shows the experimental groups with the highest to lowest range of hyperglycemia that indicates the efficacy of the treatment which can be depicted as ELD> DD > EL500 >EL 2g > reference drugs. In the table, reference drugs are SZ + insulin + losartan; DD is SZ+ SZ + insulin + losartan + *E. littorale* decoction; ELD is SZ+ *E. littorale* decoction; EL500 is SZ+ *E. littorale* 500 mg; EL2g is SZ+ *E. littorale* 2g.

**Supplementary Table 2:** Widened QRS complex during diseased and treatment.

Days	Order of widened QRS complex (ms)
**Day 10**	DD > SZ > reference drug > ELD > EL500 > EL2g
**Day 20**	SZ > reference drug > EL500 > ELD > EL2g > DD
**Day 30**	SZ > reference drug > ELD > EL2g> EL500 > DD
**Day 40**	SZ > reference drug > ELD > EL2g > EL500 > DD
**Day 50**	SZ > reference drug > EL2g > EL500 > ELD > DD
**Day 60**	SZ > reference drug > EL 500 > EL2g > DD > ELD

Supplementary Table 2 shows the experimental groups with the highest to lowest range of widened QRS complex. In SZ (group II) widened QRS complex was present from day 10 indicating the impairments of ventricular depolarization. The efficacy of the treatments to restore QRS complex can be indicated as: DD >ELD > EL500 > EL2g > reference drugs.

**Supplementary Table 3:** Elevated R-amplitude during diseased and treatment.

Days	Order of elevated R-amplitude (mV)
**Day 10**	ELD > reference drug > SZ > DD > EL2g > EL500
**Day 20**	SZ > reference drug > DD > EL500 > EL2g > ELD
**Day 30**	SZ > reference drug > EL500 > DD > EL2g > ELD
**Day 40**	DD > reference drug > EL500 > ELD > EL2g > SZ ^†^
**Day 50**	Reference drug > DD > ELD > EL2g > EL500 > SZ ^†^
**Day 60**	EL2g > reference drug > DD > ELD > EL500 > SZ ^†^

Supplementary Table 3 shows the experimental groups with the highest to lowest range of elevated R-amplitude. In SZ (group II) elevated levels were found until day 30 that may be due to the elongated cardiomyocytes. Followingly from day 40, SZ induced rats exhibited shortened R-amplitude which could be due to the loss of cardiomyocytes (indicated by ^†^) but not restoration of R-amplitude in SZ. Though all of the following groups exhibited elevated levels on day 10, their efficacy in restoring R-amplitude to near normal (neither elevated nor shortened) can be indicated as: ELD > DD > EL500 > EL2g > reference drugs.

**Supplementary Table 4:** Elevated S-T segment during diseased and treatment.

Days	Elevated S-T segment (ms)	Elevated S-T segment (mV)
**10**	ELD > SZ > reference drug > DD > EL500 > EL2g	EL500 > EL2g > ELD > reference drug > DD >SZ
**20**	SZ > ELD > reference drug > EL2g > EL500 > DD	EL500 > reference drug > ELD > EL2g > DD > SZ
**30**	SZ > EL500 > reference drug > EL2g > ELD > DD	SZ > reference drug > ELD > EL2g > DD > EL500
**40**	SZ > DD > reference drug > EL500 > EL2g > ELD	SZ > EL500 > ELD > DD > reference drug > EL2g
**50**	SZ > reference drug > DD > EL500 > EL2g > ELD	SZ > EL2g > EL500 > reference drug > ELD > DD
**60**	SZ > reference drug > ELD > EL2g > EL500 > DD	SZ > ELD > reference drug > DD> EL2g > EL500

Supplementary Table 4 shows the experimental groups with the highest to lowest range of elevated S-T segment (ms and mV). In SZ (group II) elevated durations of S-T segment (ms) were found to initiate from day 10 and become intense between days 20 to 60. The amplitude of S-T segment (mV) seems to be elevated from day 30 preceded by shortened amplitudes that are characteristics of hypertrophy (CH). Though all of the following groups exhibited impaired S-T segment on day 10, their efficacy in restoring S-T segment duration (ms) and amplitudes (mV) can be graded as: DD > ELD > EL2g > EL500 > reference drugs.

**Supplementary Table 5:** Prolonged R-R interval during diseased and treatment.

Days	Prolonged R-R interval (ms)
**10**	EL2g > SZ > reference drug > ELD > EL500 > DD
**20**	SZ > EL2g > ELD > reference drug > EL500 > DD
**30**	ELD > SZ > EL2g > EL500 > reference drug > DD
**40**	SZ > EL2g > EL500 > ELD > reference drug > DD
**50**	EL2g > SZ > reference drug > EL500 > DD > ELD
**60**	SZ > EL2g > reference drug > DD> EL500 > ELD

Supplementary table 5 shows the experimental groups with the highly prolonged R-R interval (ms) which is indirectly proportional to heart rate (HR) wherein SZ (group II) showed increased R-R interval from day 10 and beyond. Though the treatment groups exhibited impaired or less restored R-R interval at various durations, their efficacy can be indicated in the decreasing order as: DD > ELD > EL500 > EL2g > reference drugs.

Supplementary Figure 1 Concentration of blood glucose during early durations. Five different patterns of glycemic shift were observed in these till the end of 9 days (indicated in purple box) in the SZ (diseased group). SZ (group II) and SZ+ *E. axillare (littorale)* (group V) showed significant glycemic changes between hour 1 to day 9 (pink dashed lines).

Supplementary Figure 2 Body weight changes among experimental groups at various durations.

Supplementary Figure 3 ECG parameters for atrial function in experimental groups. (a and b) Atrial functions indicated by P wave (ms and mV) were less impacted due course among the experimental groups. (c) Atrioventricular communication indicated by P-R wave (ms) was affected significantly only at earlier duration (day 10).

Supplementary Figure 4 Correlation between blood glucose and ECG ventricular parameters.
Pearson’s correlation (coefficient score > 0.7) indicated that only 0^th^ day blood glucose levels were closely associated with the changes in ventricular events across the durations.

## Figures and Tables

**Table 1:** Multiple comparisons for durations.

Statistical Indication	Comparisons for durations in early blood glucose shifts (Figure 1)	Comparisons for durations in blood glucose shifts and ECG parameters (Figure 2–6 and Supplementary Figures)
**a**.	h 0 vs. h 1	day 0 vs. day 10
**b**.	h 0 vs. h 2	day 0 vs. day 20
**c**.	h 0 vs. h 3	day 0 vs. day 30
**d**.	h 0 vs. h 23	day 0 vs. day 40
**e**.	h 0 vs. h 25 (day 1)	day 0 vs. day 50
**f**.	h 0 vs. day 2	day 0 vs. day 60
**g**.	h 0 vs. day 3	--
**h**.	h 0 vs. day 4	--
**i**.	h 0 vs. day 5	--
**j**.	h 0 vs. day 6	--
**k**.	h 0 vs. day 7	--
**l**.	h 0 vs. day 8	--
**m**.	h 0 vs. day 9	--
Significance (p < 0.05) indicated by * in superscript in all the figures		

Table 1 shows the comparisons performed among different hours (h) and days.

**Table 2:** Comparison between the experimental groups.

** Multiple comparisons for groups (**Figures 1–6)
normal (group I) Vs SZ (groups II)
SZ (group II) Vs SZ+ insulin+ losartan (group III)
SZ (group II) Vs SZ+ insulin+ losartan+ *E. axillare (littorale)* decoction (group IV)
SZ (group II) Vs SZ+ *E. axillare (littorale)* decoction (group V)
day 5 SZ (group II) Vs SZ+*E. axillare (littorale)* 500 mg (group VI)
SZ (group II) Vs SZ + *E. axillare (littorale)* 2g (group VII)
Significance (p < 0.05) indicated by * in Figure 1 and ** in Figures 2 to 9

Table 2 shows the groups comparisons.
