# Supplementary figures and images for: Progression of electrophysiological impairments in diabetic cardiomyopathy and intervention using Enicostemma axillare
Source: Turk J Biol. 2024 Nov 4;49(2):148–61. doi: 10.55730/1300-0152.2733 (PMC12068667; doi:10.55730/1300-0152.2733)

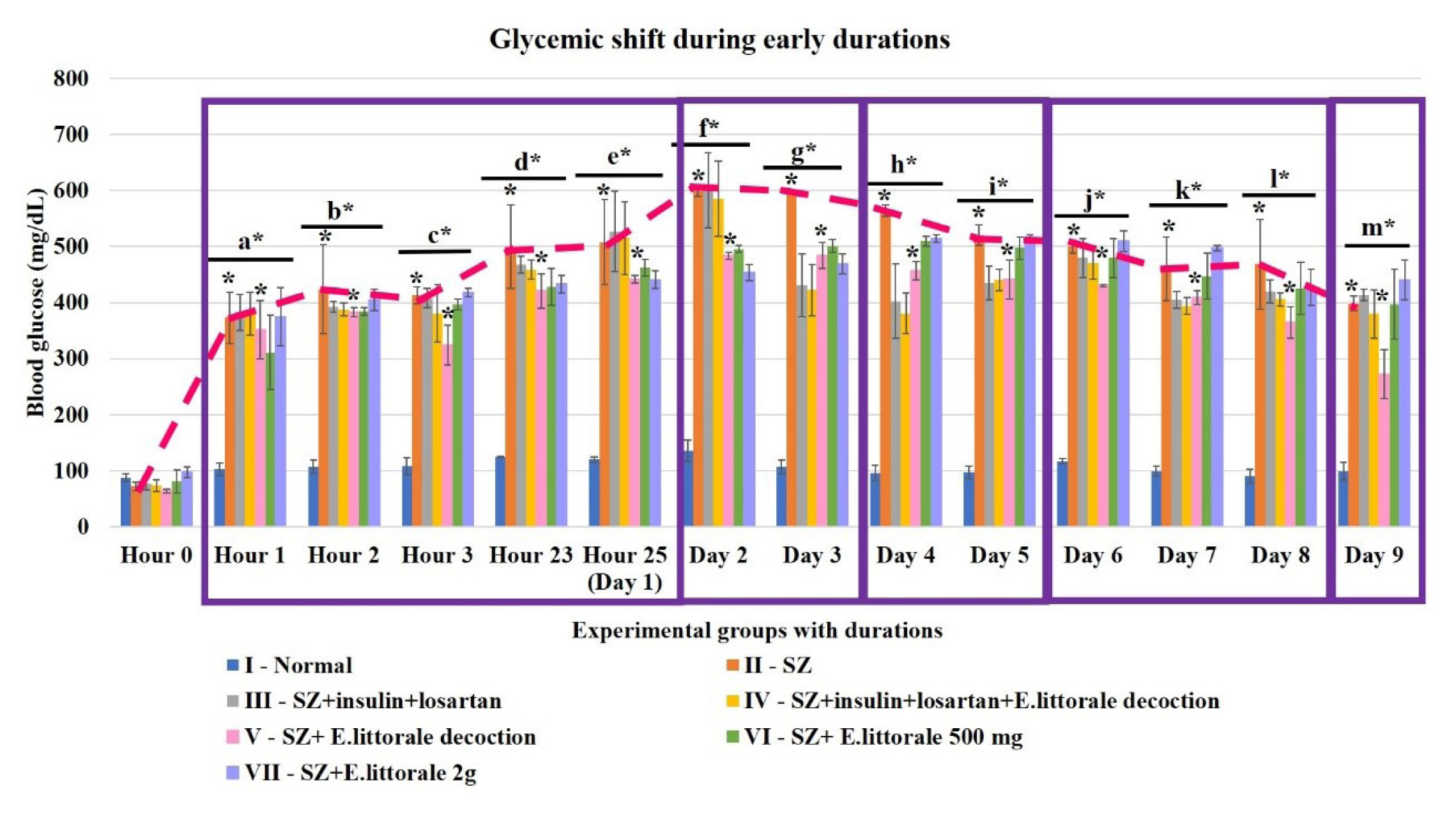

Supplement: Supplementary Figure 1 — Concentration of blood glucose during early durations. Five different patterns of glycemic shift were observed in these till the end of 9 days (indicated in purple box) in the SZ (diseased group). SZ (group II) and SZ+ E. axillare (littorale) (group V) showed significant glycemic changes between hour 1 to day 9 (pink dashed lines). [file tjb-49-02-148s1.tif]

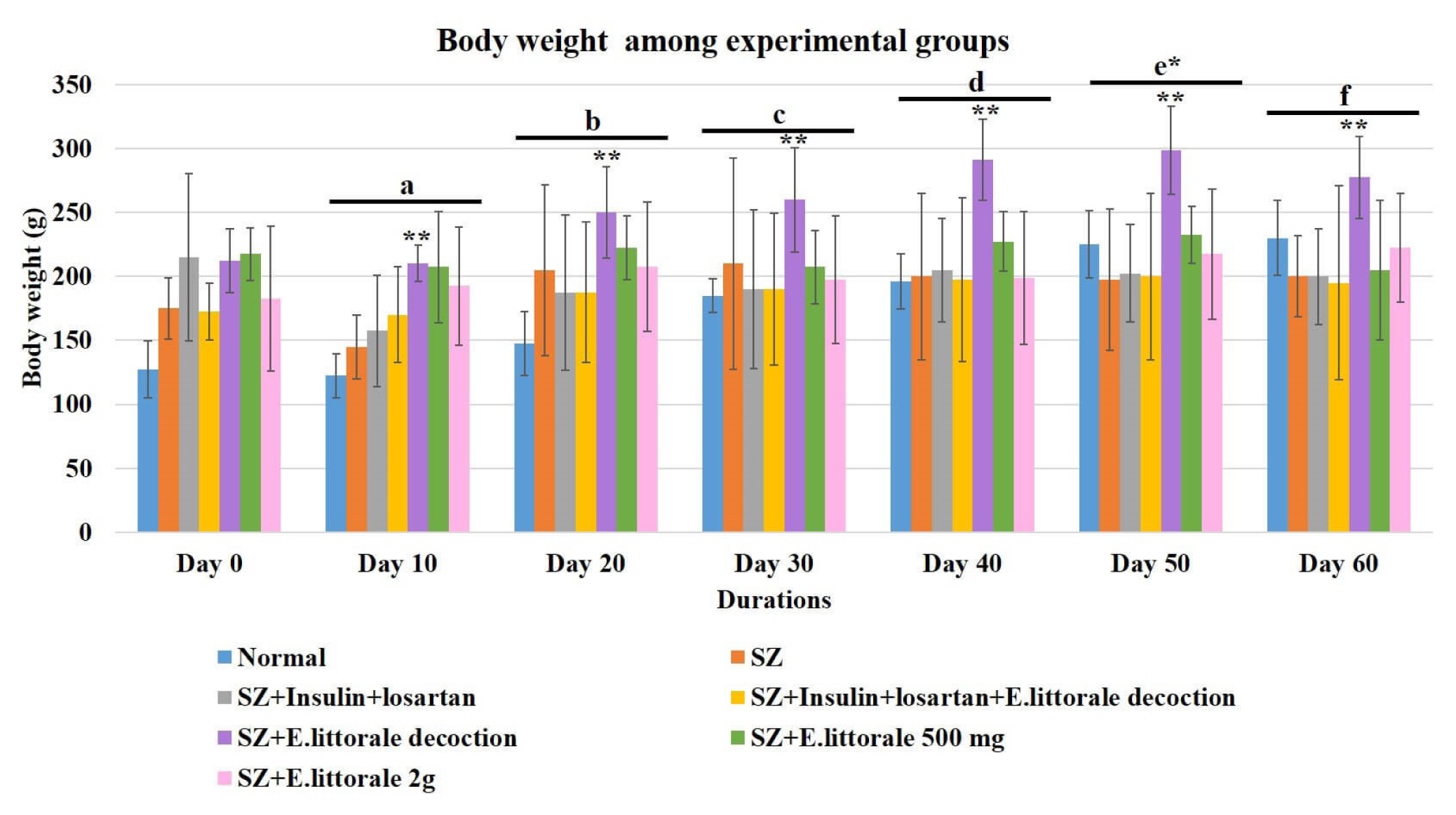

Supplement: Supplementary Figure 2 — Body weight changes among experimental groups at various durations. [file tjb-49-02-148s2.tif]

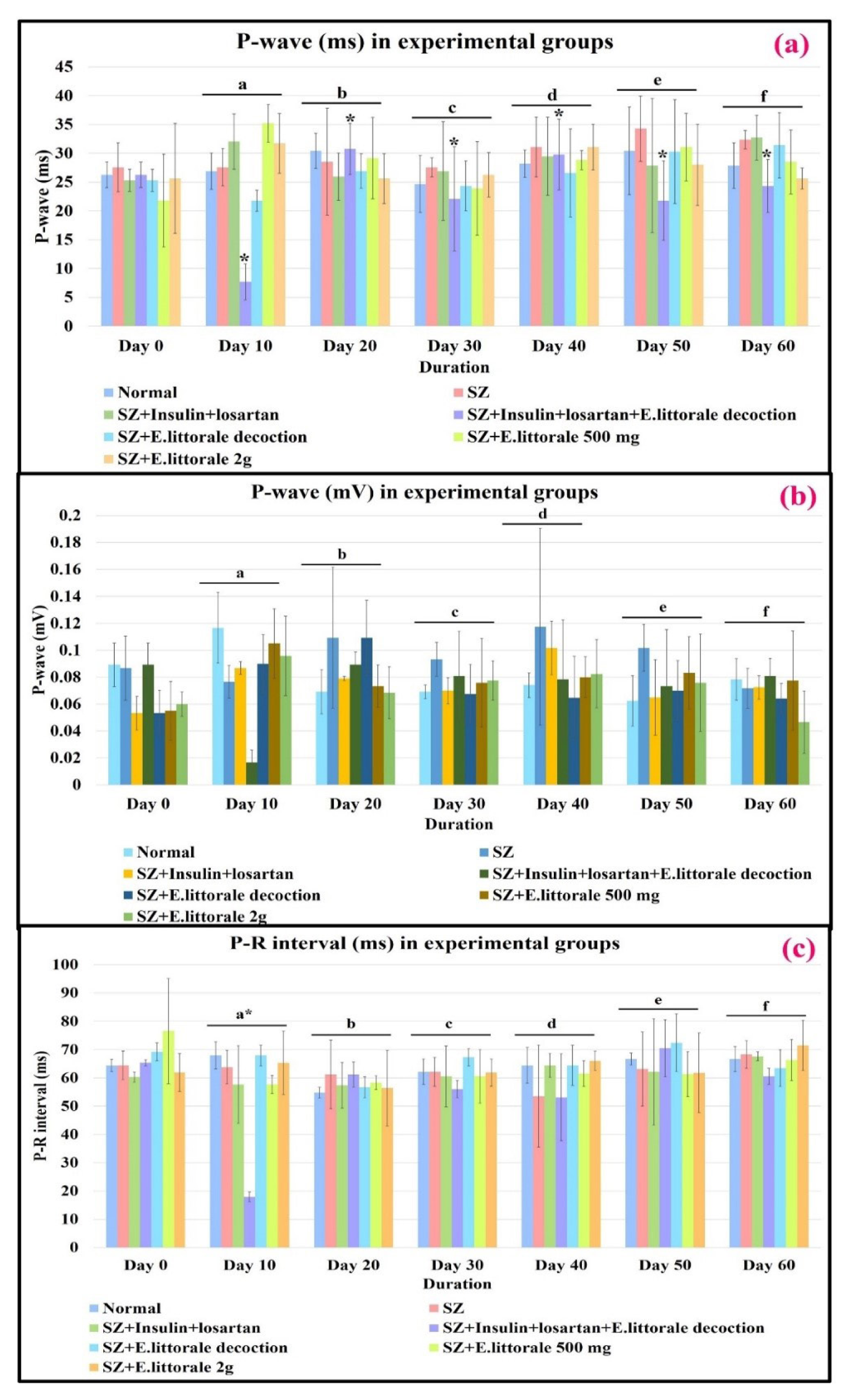

Supplement: Supplementary Figure 3 — ECG parameters for atrial function in experimental groups. (a and b) Atrial functions indicated by P wave (ms and mV) were less impacted due course among the experimental groups. (c) Atrioventricular communication indicated by P-R wave (ms) was affected significantly only at earlier duration (day 10). [file tjb-49-02-148s3.tif]

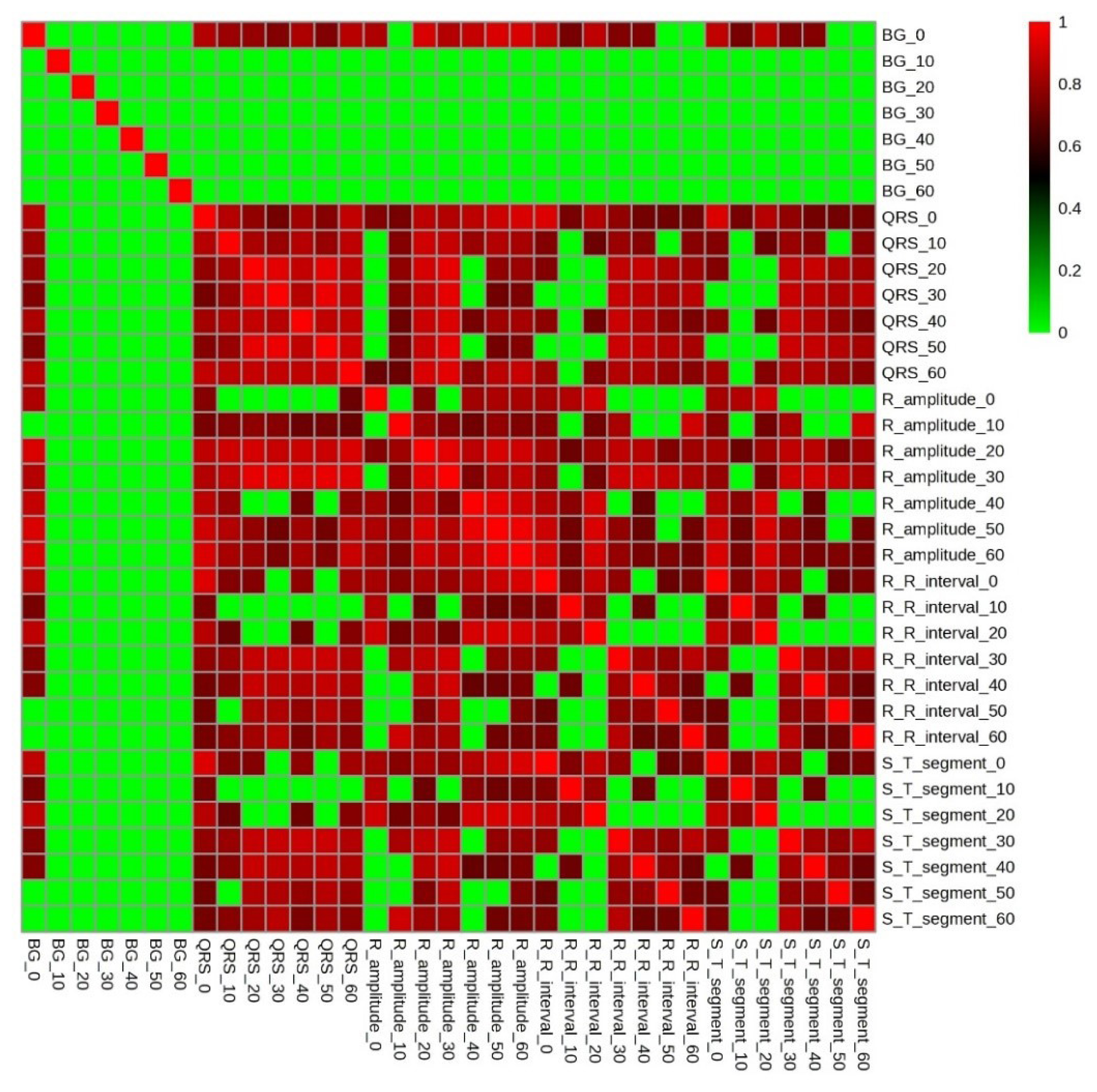

Supplement: Supplementary Figure 4 — Correlation between blood glucose and ECG ventricular parameters. Pearson’s correlation (coefficient score > 0.7) indicated that only 0th day blood glucose levels were closely associated with the changes in ventricular events across the durations. [file tjb-49-02-148s4.tif]
